# Supplementary material for: Mechanism of Synergistic Photoinactivation Utilizing Curcumin and Lauric Arginate Ethyl Ester against Escherichia coli and Listeria innocua
Source: Foods. 2023 Nov 21;12(23):4195. doi: 10.3390/foods12234195 (PMC10706742; doi:10.3390/foods12234195)
Supplement: Supplementary file 1 [file foods-12-04195-s001.zip › foods-2699308-supplementary.pdf]

Supplementary Figure S1: Absorbance spectra of diluted curcumin-LAE micelle and its components

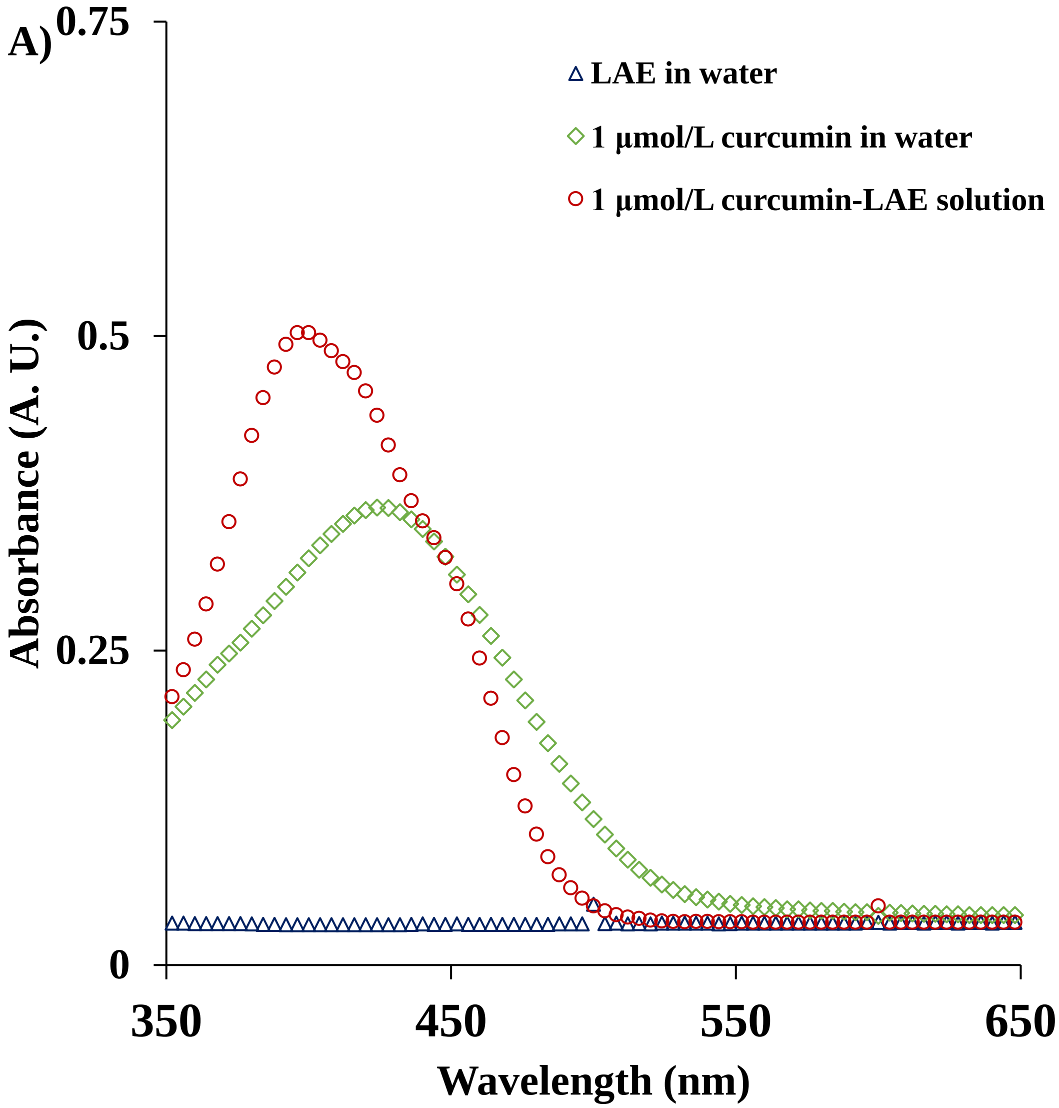

Supplementary Figure S1. Absorbance spectra of the diluted LAE micelles, unencapsulated curcumin in pH 3.5 water, and curcumin-LAE solution after 10 min.

Supplementary Figure S2: Stability of diluted curcumn-LAE micelle

A)

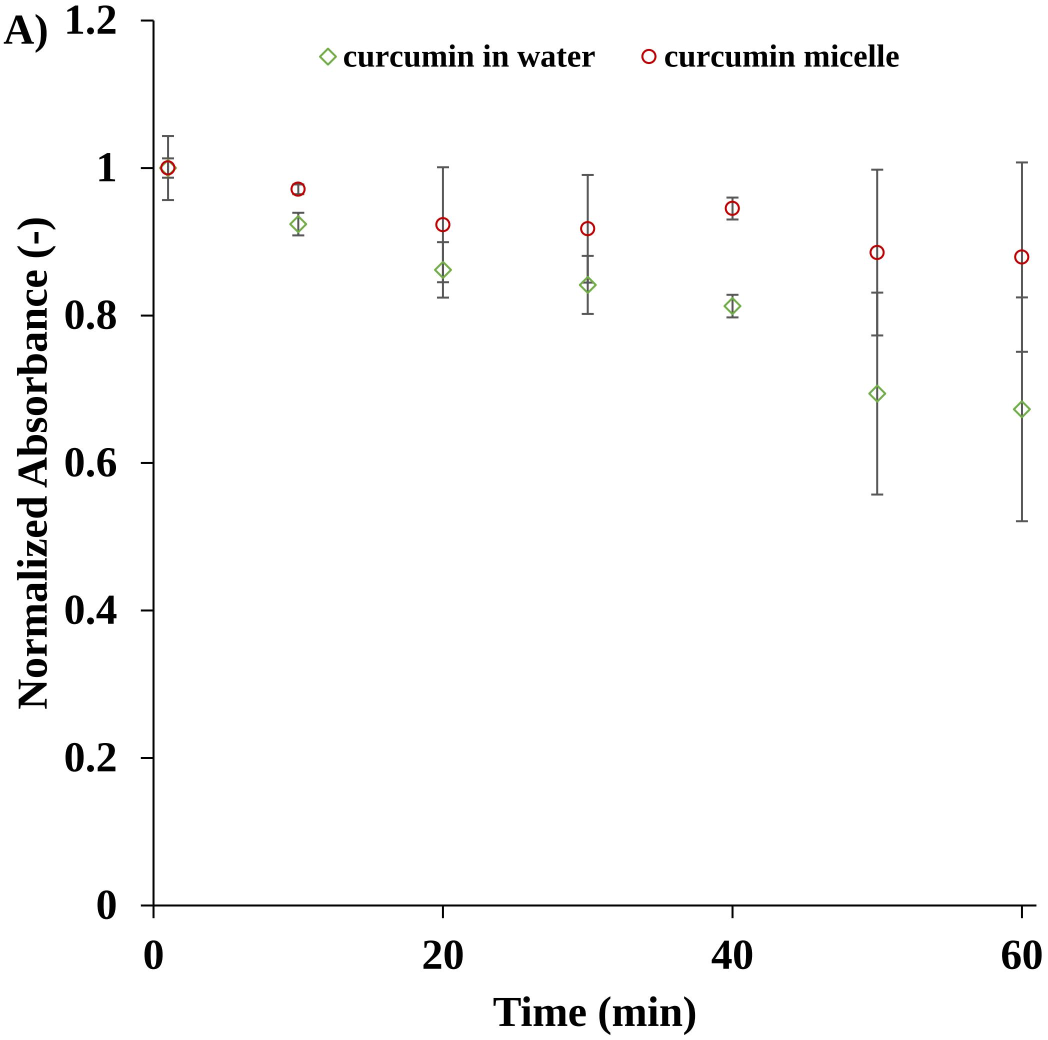

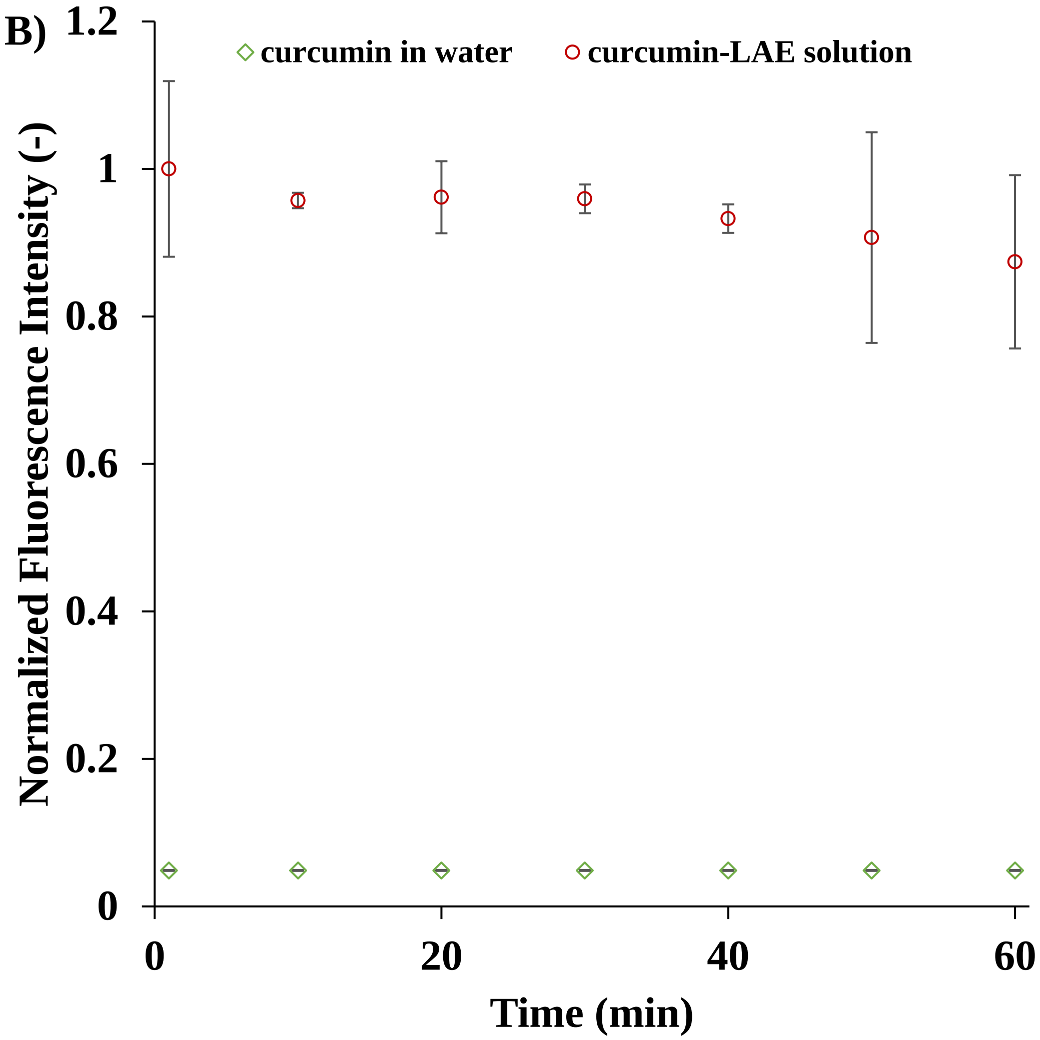

Supplementary Figure S2. A) Normalized absorbance (at 400 nm) and B) fluorescence emission intensity (λ_{exc}=365 nm, λ_{em}= 540 nm) of diluted curcumin and curcumin-LAE solutions in water at pH 3.5 as a function of time.

Supplementary Figure S3: SEM micrograph of treated *L. innocua* cocktail

A)

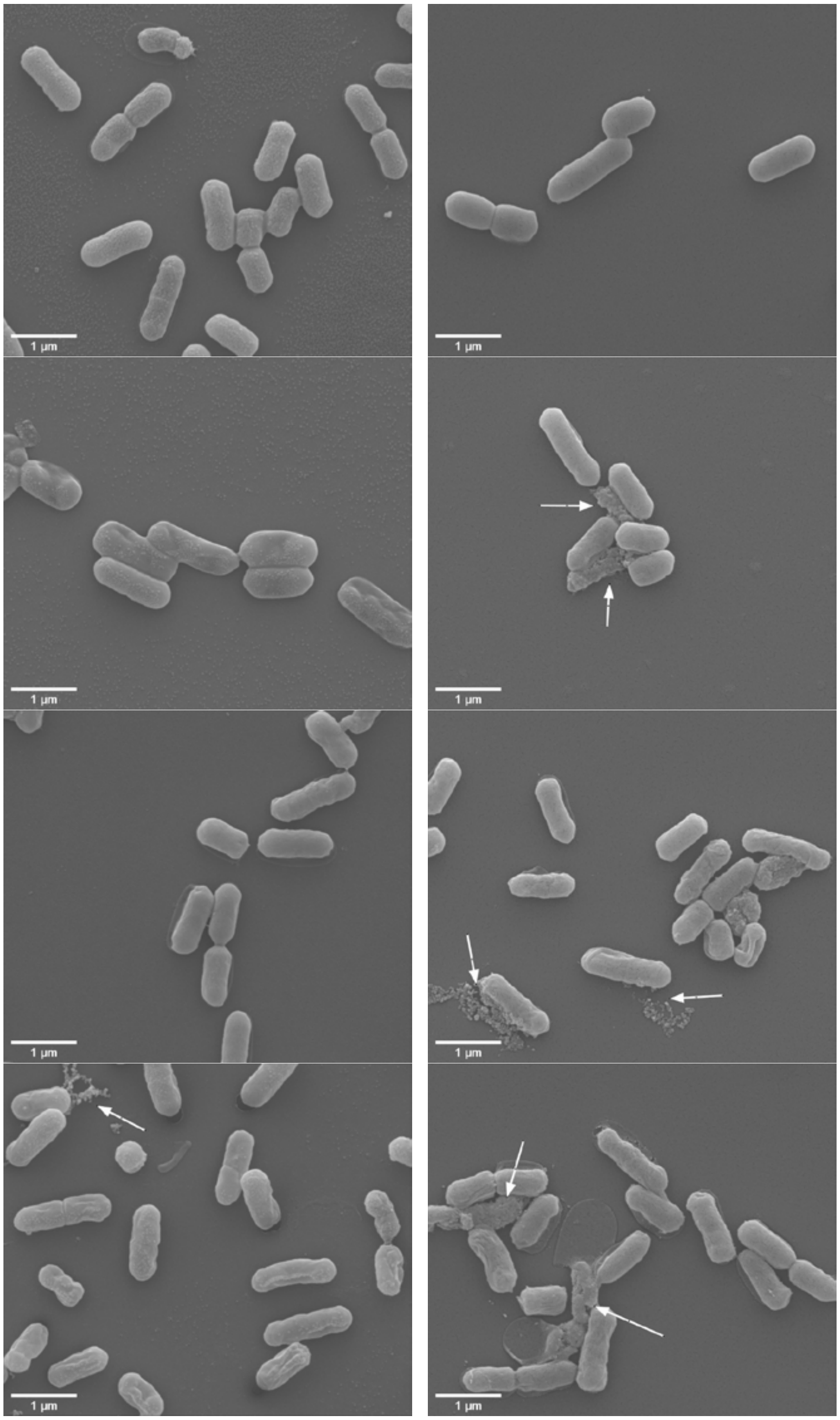

B)

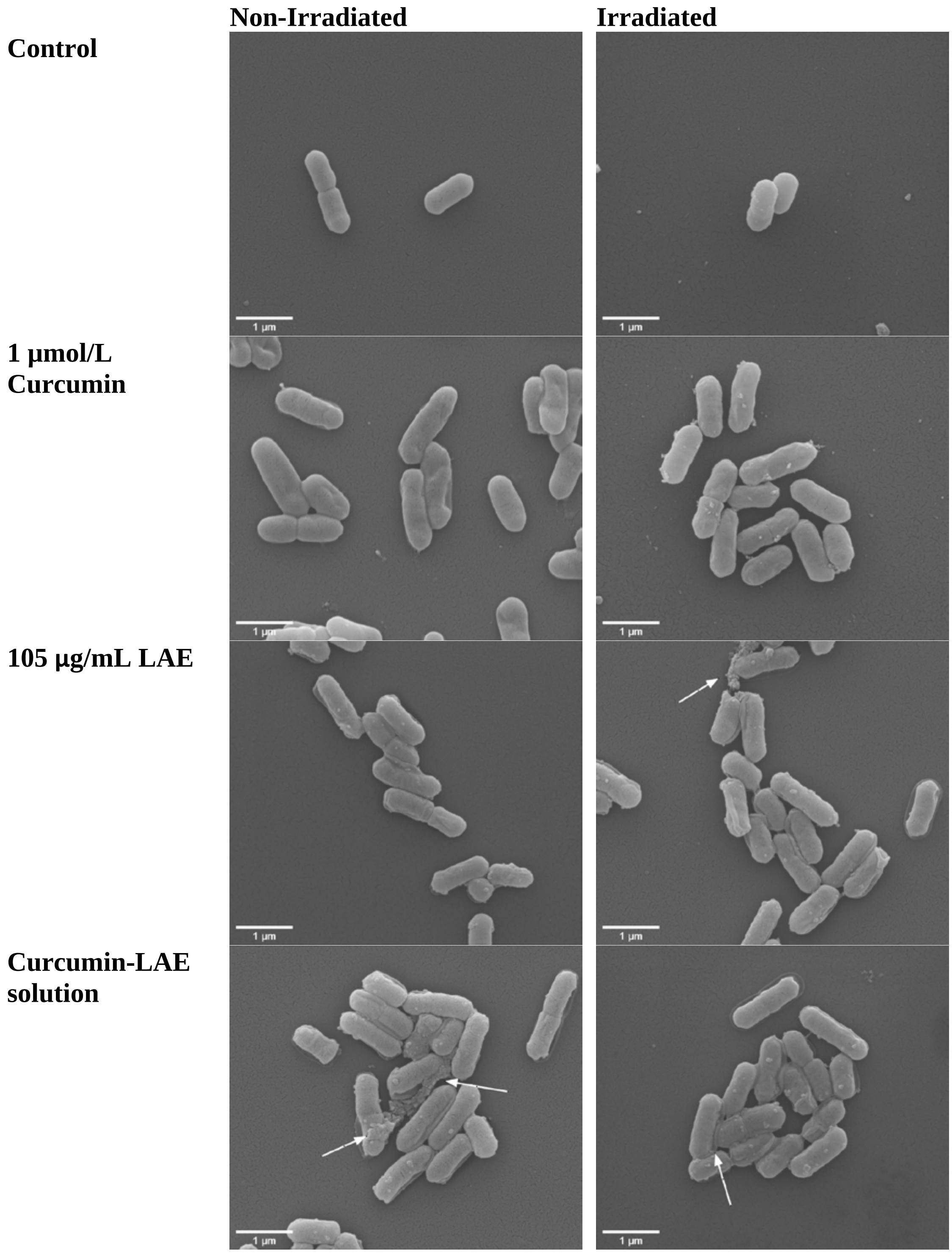

Supplementary Figure S3. SEM micrograph of non-irradiated and irradiated cocktail of *L. innocua* treated with curcumin, LAE, curcumin-LAE solutions at A) pH 3.5 and B) pH 7. The white arrows indicate damage and leakage from the cell.

Supplementary Figure S4: TEM micrograph of treated *L. innocua* cocktail

A)

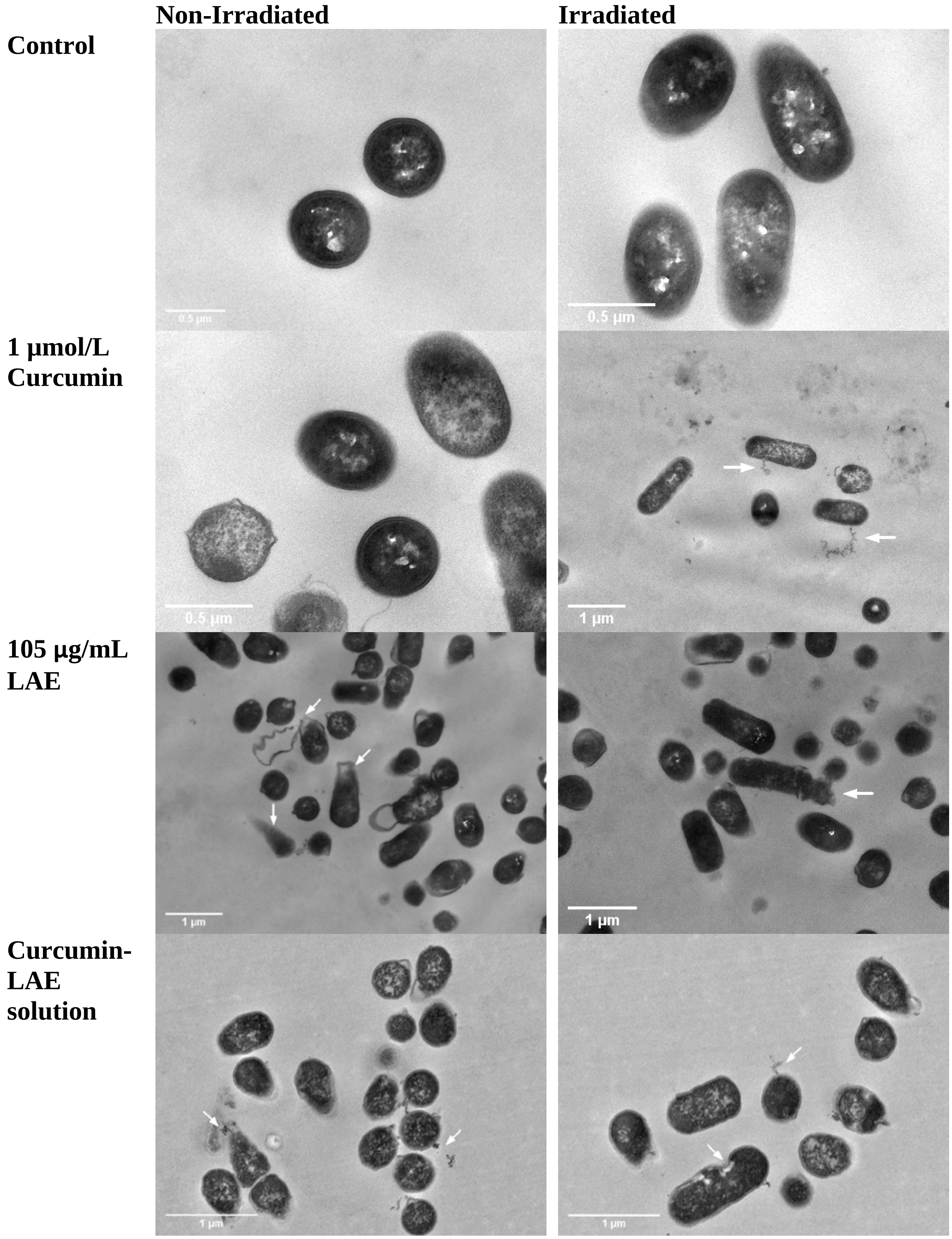

B)

Non-Irradiated **Irradiated**

Control

1 µmol/L Curcumin

105 µg/mL LAE

Curcumin-LAE solution

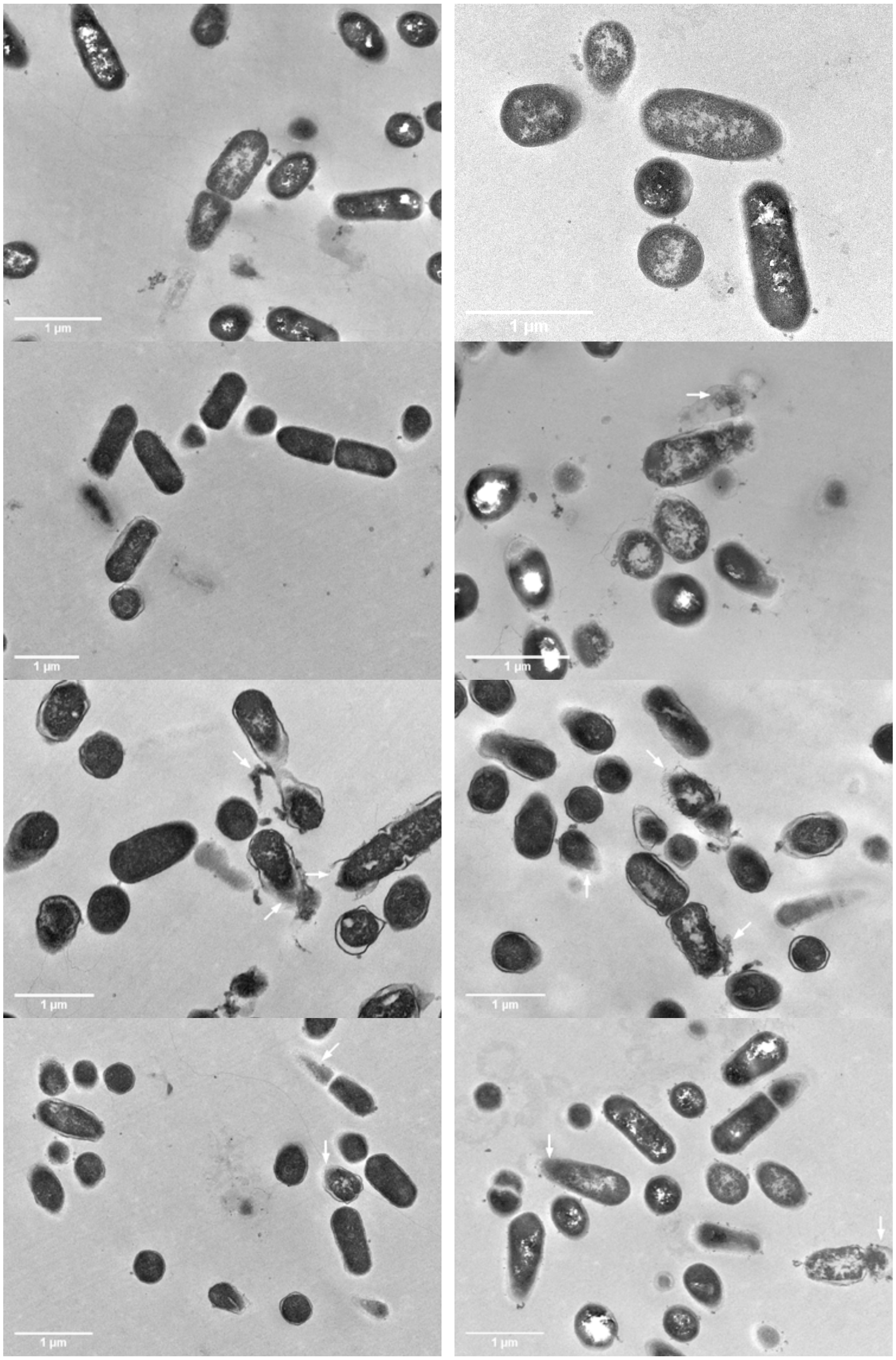

Supplementary Figure S4. TEM micrograph of non-irradiated and irradiated cocktail of *L. innocua* treated with curcumin, LAE, curcumin-LAE solutions at A) pH 3.5 and B) pH 7. The white arrows indicate dissolution of cellular membrane and leakage from the cell.

Supplementary Figure S5: live/dead cell assay of treated *L. innocua* cocktail

A)

NON-IRRADIATED	IRRADIATED	NON-IRRADIATED	IRRADIATED
Control	Control	1 µmol/L Curcumin	1 µmol/L Curcumin

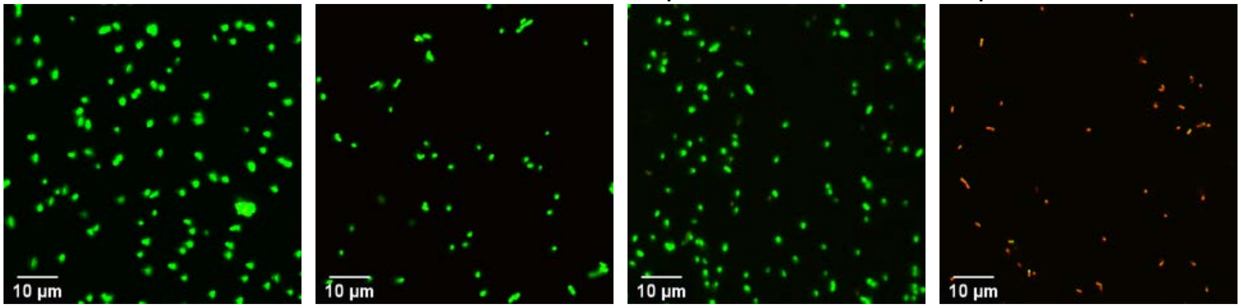

**0.5 ± 0.8**	**4.1 ± 4.3**	**4.4 ± 2.6**	**98.9 ± 2.6**
105 µg/mL LAE	105 µg/mL LAE	Curcumin-LAE solution	Curcumin-LAE solution

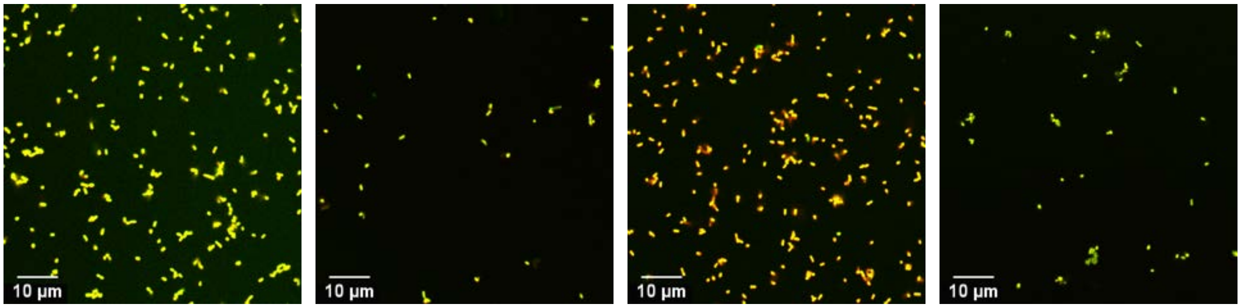

**97.8 ± 2.1**	**95.7 ± 5.9**	**100**	**98.6 ± 3.1**

B)

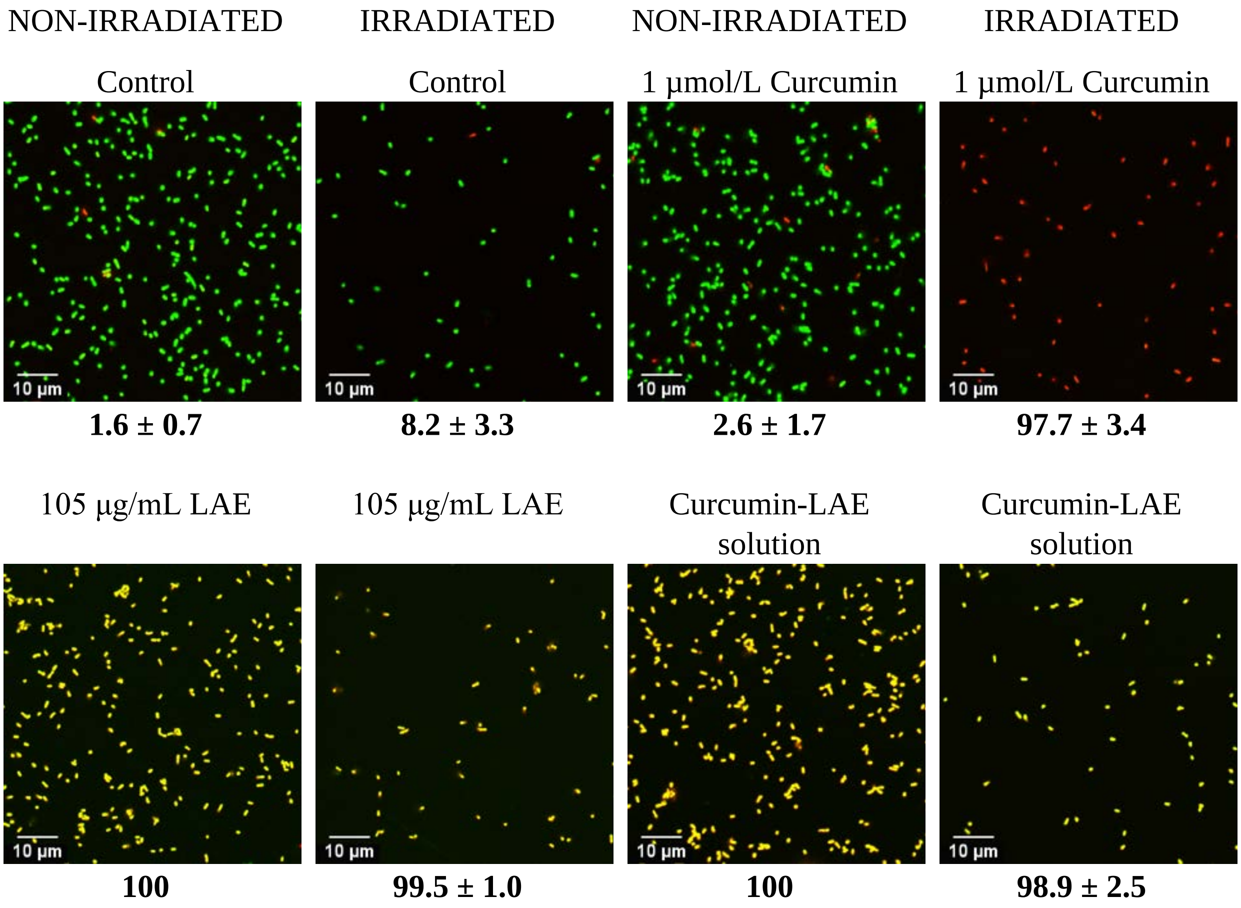

Supplementary Figure S5. Micrographs of non-irradiated and irradiated cocktail of *L. innocua* treated with curcumin, LAE, curcumin-LAE solutions at A) pH 3.5 and B) pH 7, stained with Syto9 (green) and propidium iodide (yellow, red). The percentage of cocktail of *L. innocua* at pH 3.5 or pH 7 with permeable membranes (yellow, red) is listed below each micrograph.
